# Identification and functional characterization of a novel *Acinetobacter pittii* bacteriophage-encoded depolymerase

**DOI:** 10.3389/fcimb.2025.1608526

**Published:** 2025-09-12

**Authors:** Na Zhang, Wei Li, Xue Du, Danish Daniyal, Meng-ai Feng, Jiaoyang Xu, Ziqin Yang, Hailin Jiang, Muhammad Sheraz, Honglan Huang, Santasree Banerjee, Hongyan Shi

**Affiliations:** ^1^ Department of Pathogen Biology, College of Basic Medical Sciences, Jilin University, Changchun, Jilin, China; ^2^ Department of Genetics, Qujing Maternal and Child Health-care Hospital, Qujing, Yunnan, China; ^3^ Department of Clinical Laboratory, China-Japan Union Hospital of Jilin University, Changchun, Jilin, China; ^4^ Clinical Laboratory, Affiliated Hospital of Changchun University of Chinese Medicine, Changchun, China; ^5^ Department of Genetics, College of Basic Medical Sciences, Jilin University, Changchun, China

**Keywords:** *Acinetobacter pittii*, bacteriophage, depolymerase, antibacterial activity, biofilm, antibiotic combination

## Abstract

**Introduction:**

*Acinetobacter pittii* is increasingly recognized as a significant cause of nosocomial infections. Bacteriophage-encoded depolymerases that degrade capsular polysaccharides (CPS)—a major virulence factor of *A. pittii*—represent promising therapeutic tools.

**Methods:**

This study identified and characterized a novel depolymerase, designated 31TSP, derived from the *A. pittii* bacteriophage 31Y. Its functional stability across various pH levels (5–11) and temperatures (4 °C to 121 °C) was assessed. The inhibitory effect of 31TSP on biofilm formation and its disruptive activity against preformed biofilms were evaluated using crystal violet staining, viable cell counts and scanning electron microscopy. Combinatorial treatments with 31TSP and ampicillin were conducted. Furthermore, the enzyme's stability under different ion concentrations (NaCl) and its ability to enhance serum bactericidal activity were tested under experimental conditions.

**Results:**

Characterization demonstrated that 31TSP exhibits a broad host range against *A. pittii*, *A. baumannii*, and *A. nosocomialis*. The enzyme degraded the CPS of host bacteria and displayed inhibition effects on sensitive hosts. 31TSP retained functional stability across a wide pH range (5–11) and temperatures from 4 °C to 121 °C. Its inhibitory effect on biofilm formation and disruptive activity against preformed biofilms were confirmed. Notably, combinatorial treatment with 31TSP and ampicillin significantly enhanced biofilm inhibition and disruption at 24 hours post-treatment. However, 31TSP did not maintain stability under different ion concentrations (NaCl) and could not enhance serum bactericidal activity under the experimental conditions.

**Discussion:**

These findings support the potential of 31TSP as an antibacterial agent against Acinetobacter infections. The observed synergy with conventional antibiotics, such as ampicillin, suggests a promising combinatorial strategy for future therapeutics targeting *Acinetobacter*. The enzyme's stability under extreme conditions of temperature and pH further underscores its therapeutic potential. However, its instability in varying ionic environments and lack of serum bactericidal enhancement highlight aspects requiring further investigation for clinical application.

## 1 Introduction

The *Acinetobacter calcoaceticus-baumannii* (ACB) complex has emerged as a significant nosocomial pathogen (Lee et al., 2021; Liu et al., 2015), causing diverse human infections including pneumonia, bacteremia, wound infections, meningitis, and urinary tract infections (Chusri et al., 2014). However, routine clinical diagnostics lack specificity for distinguishing ACB complex species (Vijayakumar et al., 2019). This limitation has historically led to underestimation of non-*baumannii* species in clinical settings (Pailhoriès et al., 2018).

Advances in molecular identification techniques (Teixeira et al., 2017; Marí-Almirall et al., 2019) and increased clinical isolation have established *A. pittii* as a prominent nosocomial pathogen (Chusri et al., 2014). Notably, carbapenem-resistant *A. pittii* (CRAP) strains have disseminated globally (Pailhoriès et al., 2018; Iimura et al., 2020; Tian et al., 2022), with rising antibiotic resistance rates (Zhu and Mathur, 2022). These trends necessitate urgent development of novel therapeutic agents against *A. pittii* infections.

Bacteriophages (phages) are natural co-evolutionary partners of bacteria, functioning as targeted antimicrobial agents through host-specific infection (Chen et al., 2022). However, their narrow host range—while advantageous for specificity—poses challenges for clinical implementation, necessitating precise strain-matched selection for therapeutic applications (Ringaci et al., 2022). Consequently, phage-derived enzymes (e.g., endolysins, depolymerases, and holins) have emerged as promising antibacterial candidates (Pires et al., 2016; Yuan et al., 2021).

Depolymerases are of particular interest due to their ability to degrade bacterial capsular polysaccharides (CPS), a major surface-exposed virulence factor. CPS serves as a protective barrier that facilitates bacterial adhesion, impedes antibiotic penetration, and confers resistance to phagocytosis and opsonization by host immune cells (Paczosa and Mecsas, 2016). CPS degradation sensitizes bacteria to immune-mediated clearance (Chen et al., 2022). Genes encoding depolymerases are typically situated near structural protein genes (e.g., tail fibers, tail sheath, baseplate, and neck connector proteins) in phage genomes and may occasionally share open reading frames (ORFs) with these genes (Cai et al., 2020). Bacteriophage-encoded depolymerases have been demonstrated to attenuate bacterial virulence in multiple pathogens, including *Escherichia coli* (Chen et al., 2020), *Klebsiella pneumoniae* (Li et al., 2021; Cui et al., 2023), *A. baumannii* (Liu et al., 2019a), and *Proteus mirabilis* (Rice et al., 2021). However, research on depolymerase-mediated virulence reduction remains limited for *A. pittii* (Domingues et al, 2021). This gap underscores the need to identify and characterize additional depolymerases targeting *A. pittii* strains.

In this study, we identified an enzyme, TSP, capable of detaching *A. pittii* Ap31 CPS. We determined its activity under different pH values, temperatures, and ion concentrations. Additionally, we conducted research on the combined use of TSP and antibiotics against biofilms, as well as serum sensitivity assays mediated by TSP. Our findings indicate the applicability of bacteriophage-encoded capsule depolymerases as a novel strategy to control drug-resistant *A. pittii* infections.

## 2 Materials and methods

### 2.1 *Acinetobacter pittii* strain and the isolation of bacteriophage

All *Acinetobacter* strains employed in this study were isolated from the affiliated hospital of Changchun University of Chinese Medicine. Routine cultivation of these strains was conducted at 37 °C in LB broth or on LB agar (1.5% [wt/vol] agar).

Using *A. pittii* Ap31 as the host, bacteriophages were isolated from wastewater obtained from the First Hospital of Jilin University. Initially, the wastewater was centrifuged at 10,000 rpm for 10 minutes, and the supernatant was filtered through a 0.22 μm filter. The filtered supernatant was added to a 100 mL mixed bacterial culture in a logarithmic phase, and the mixture was incubated overnight at 160 rpm and 37 °C. The following day, the culture was centrifuged at 10,000 rpm for 10 minutes, and the supernatant was filtered through a 0.22 μm, 10 μL filter. A 10μL aliquot was then spotted onto LB plates overlaid with the corresponding bacterial strain to detect bacteriophage plaques. The bacteriophage was purified by repeating the double agar overlay method four times Bacteriophage titers were assessed using the double-layer agar method as described previously (Kropinski et al., 2009). The one step grow curve was tested according to standard protocol for therapeutic phage preparation (Luong et al., 2020).

### 2.2 Observation of morphology through transmission electron microscopy

Bacteriophage particles were diluted 20 times, and 30 μL of the diluted solution was dropped onto a 400-mesh copper grid. After adsorption for 15 minutes, the excess liquid was removed from the side. The sample was prepared for transmission electron microscopy (TEM). Negative staining was performed using 0.2% phosphotungstic acid for 30 seconds, excess liquid was removed, and the sample was air-dried for 1 hour. The specimen was then placed on the observation stage of the HT-7800 TEM (Hitachi, Japan) and bacteriophage morphology was observed and photographed under a voltage of 100 kV.

### 2.3 Preparation and sequencing analysis of bacteriophage genomes

As previously mentioned, bacteriophage genomic DNA was extracted using the phenol-chloroform method with slight modifications (Liu et al., 2013). Briefly, 500 μL of bacteriophage particles were treated at 37°C with a final concentration of 1 μg/mL DNase I and RNase A for 1 hour to remove bacterial nucleic acids. Subsequently, 25 μL of 0.5 M EDTA (pH 8.0), 25 μL of 1 mg/mL proteinase K, and 20 μL of 10% SDS were added, followed by incubation at 56°C for 1 hour. After cooling to room temperature, bacteriophage genomic DNA was extracted using the phenol-chloroform method and then precipitated with ethanol. After thorough air-drying at room temperature, the precipitate was dissolved completely in 20-50 μL nuclease-free water and stored at -20°C.

The extracted bacteriophage genomic DNA was sent to Nanjing Parsona Genomics Technology Co., Ltd. for genome sequencing using the Illumina NovaSeq high-throughput sequencing platform. GeneMarkS software was employed for the analysis and prediction of open reading frames (ORFs) in the bacteriophage genome. HHpred (Söding et al., 2005) was used for remote protein homology detection and structure prediction of bacteriophage-encoded depolymerase ORFs. AlphaFold 2.0 (Jumper et al., 2021) was utilized for tertiary structure prediction with default settings, and PyMOL 2.5 was used for visualization.

### 2.4 Plasmid construction

Plasmid construction was performed using standard cloning methods. The ORF43 gene encoding the tail spike protein of bacteriophage 31Y, designated as 31TSP, was synthesized by Nanjing Parsona Genomics Technology Co., Ltd. It was then cloned into the pET28a plasmid using BamHI and HindIII restriction sites. The C-terminus of the protein was tagged with a 6×His tag.

### 2.5 Protein expression, purification, and identification

The constructed plasmid was transformed into *Escherichia coli* BL21 (DE3) cells. Recombinant plasmid-carrying *E. coli* BL21 cells were induced with 1 mM isopropyl-β-d-thiogalactopyranoside (IPTG) at 37 °C for 4 hours. Cells were harvested by centrifugation for subsequent protein purification. Ni-Berpharose FF (Beijing Borsodex Technology Co., Ltd.) was employed for nickel affinity chromatography to purify the enzyme. Briefly, the harvested cells were resuspended in sonication lysis buffer, and the cell suspension was lysed by sonication (20 minutes, 1 second/2 seconds). After centrifugation, the supernatant was collected. Then the supernatant was sequentially filtered through a 100 kDa 15 mL molecular weight cut-off (MWCO) centrifugal filter (Millipore) by centrifugation at 5,000 × g for 8 min at 4 °C. The filtrate was collected and subsequently processed through a 20 kDa MWCO centrifugal filter (Millipore) under identical centrifugation conditions. Following centrifugation, proteins retained on the membrane were eluted. The eluted proteins were loaded onto Ni-Berpharose FF. The bound proteins were eluted with an imidazole gradient ranging from 10 to 500 mM. Proteins eluted with the imidazole gradient were collected, and the molecular weight of the depolymerase was assessed using sodium dodecyl sulfate-polyacrylamide gel electrophoresis (SDS-PAGE) (Chen et al., 2022). The concentration of the depolymerase was determined using the BCA Protein Assay Kit (Beyotime) (Walmagh et al., 2013).

Depolymerase activity was determined by spot assay (Hsieh et al., 2017). LB agar plates were inoculated with 200 μL of fresh bacterial culture. After air-drying at room temperature, 5 μL of purified depolymerase was spotted on the plates. Following overnight incubation at 37°C, the formation of translucent spots on the plates was observed. Based on the imidazole concentrations present in the various depolymerase concentration experiments, we examined the antibacterial effects of different concentrations of imidazole on *A. baumannii* using the same methodology.

Additionally, antibacterial activity of depolymerase 31TSP was assessed through viable cell counting. The depolymerase was added to logarithmic-phase host bacterial cultures at final concentrations of 100 μg/mL, 50 μg/mL, and 25 μg/mL. PBS of the same volume was added as a control. After incubation at 37 °C for 1, 2, 3, 4, 5, and 6 hours, each group was serially diluted tenfold, and viable cell counts were performed. The experiment was repeated three times, and the data were presented as bacterial reduction counts (control group - treatment group).

### 2.6 Determination of the host range of the phage and capsule depolymerase

A spot assay (Hsieh., et al. 2017) was conducted by observing 100 strains of Acinetobacter bacteria stored in the laboratory to ascertain the host range of Bacteriophage 31Y and Depolymerase 31TSP.

### 2.7 Quantitative assay of depolymerase activity

Depolymerase activity was quantitatively determined by measuring the release of reducing sugars during the reaction with 3,5-dinitrosalicylic acid (DNS). Initially, bacterial capsular polysaccharide (CPS) was extracted using the Bacterial Polysaccharide Extraction Kit (Solarbio, EX1750). Strain Ap31 was cultured on LB agar plates supplemented with 0.5% glucose at 37°C for 5 days. Cells were then scraped off with 2.5 mL of 0.9% (w/v) NaCl solution, and the harvested cells were used for CPS extraction according to the kit instructions (Oliveira et al., 2021). The freeze-dried CPS powder was re-suspended in PBS to a final concentration of 2 mg/mL and incubated with 100 μg/mL of 31TSP at 37°C for 1 hour, with PBS serving as control in place of CPS or 31TSP. DNS reagent (Solarbio, D7800) was added immediately at twice the volume in each reaction mixture, followed by a 5-minute reaction at 100°C. Absorbance was measured at 540 nm (Liu et al., 2019a). All experiments were independently conducted three times.

### 2.8 Hemolysis assay

The impact of 31TSP on human red blood cell (RBC) hemolysis was assessed using a modified version of a previously described method (Chen et al., 2022). Isolated RBCs were washed twice with PBS (2,000 rpm, 10 min), diluted in PBS to a 5% (v/v) concentration, and then incubated with 31TSP at 37°C for 1 hour, with PBS and 0.1% Triton X-100-treated RBCs serving as negative and positive controls, respectively. After centrifugation at 8,000 rpm for 10 min, 100 μL of the supernatant was transferred to a 96-well microplate, and 100 μL of PBS was added to each well, °Cresulting in a final volume of 200 μL. Hemoglobin levels were measured at 540 nm. All experiments were independently conducted three times.

### 2.9 Cytotoxicity analysis of 31TSP

HEK293 cells (human embryonic kidney cells) were cultured in DMEM (Gibco) supplemented with 10% FBS (HyClone) at 37°C under 5% CO_2_ and 90% humidity for 2 days. Subsequently, the cells were trypsinized and seeded into 96-well plates at a density of 10^4^ cells per well and incubated at 37°C for 24 h. Then, the medium was replaced by 200 μL new medium containing 31TSP (50 μg/mL). After 12 h incubation, 10 μL WST-8 solutions were added into each well for another 2 h incubation at 37°C. The cell viability was evaluated by absorbance at 450 nm using a microplate reader (Tecan). Three independent experimental repeats were performed, each with triplicate wells per group including PBS controls.

### 2.10 Depolymerase stability analysis

Stability analysis of the depolymerase was conducted following a modified version of the method described in a previous study(Oliveira et al., 2019). To evaluate the pH stability of 31TSP, phosphate-buffered saline (PBS) solutions were adjusted to pH values ranging from 1 to 14 using HCl or NaOH. For each pH condition, 31TSP (final concentration: 100 μg/mL) and 100 μL of log-phase bacterial culture were added into 1.8 mL of the corresponding PBS. The total reaction volume was adjusted to 2 mL using PBS at the same pH. Control groups contained only PBS (at respective pH values) and an equivalent volume of bacterial suspension. All samples were incubated at 37°C with shaking for 3 h. After incubation, serial dilutions were performed, followed by overnight plating for colony counting. The antimicrobial activity of 31TSP at different pH levels was determined by calculating the reduction in bacterial counts (CFU/mL) relative to the corresponding pH-matched control (Δlog reduction). A higher reduction value indicates greater enzymatic stability and activity under the tested pH conditions.

For temperature stability, 31TSP was respectively treated at temperatures of 4, 25, 37, 50, 60, 70, 80, 90, 100, and 121°C for 30 minutes. After cooling to room temperature, 31TSP was mixed with the host bacterial culture to achieve a final concentration of 100 μg/mL. Same volume of PBS was added into bacteria culture as control. All samples were incubated at 37°C with shaking for 3 h. Then the living bacteria were cultured and counted as in pH stability test.

To assess the effect of ionic strength on 31TSP activity, log-phase bacterial culture was mixed with 31TSP at a final concentration of 100 μg/mL. The ion concentration of the mixture was then adjusted by adding 1 M NaCl to achieve final NaCl concentrations of 50 μM, 150 μM, 300 μM, and 500 μM. Negative control was prepared by adding an equivalent volume of sterile distilled water (ddH_2_O) instead of NaCl (0 μM). All samples were incubated at 37°C with shaking for 3 h. Then the living bacteria were cultured and counted as in pH stability test.

### 2.11 Serum killing assay

The optimal volume ratio of serum to enzyme-treated bacteria was determined as described previously (Liu et al., 2019b). Overnight cultured host bacteria were co-incubated with or without 100 μg/mL TSP at 37 °C for 3 hours. The enzyme-treated host bacteria were added to healthy human serum at volume ratios of 3:1, 1:1, or 1:3, with a final reaction volume of 200 μL. After incubation at 37 °C for 3 hours, bacterial viability was determined by colony counting. The experiment was independently conducted three times.

To assess the role of serum complement in the serum bactericidal assay, a strain sensitive to Bacteriophage 31Y and 31TSP (*A. pittii* Ap30) and a strain insensitive (*A. baumannii* Ab5) to 31Y and 31TSP were also selected (Chen et al., 2022). Bacteria were treated with enzyme for 3 hours at 37°C and then mixed with active or heat-inactivated serum (56°C, 30 minutes) at a 3:1 volume ratio. After incubation at 37°C for 3 hours, bacterial viability was determined by colony counting. Untreated bacteria, along with equal volumes of PBS, 31TSP, active and heat-inactivated serum, were cultured as controls, and bacterial viability was determined after 6 hours of incubation at 37°C. All experiments were independently conducted three times.

### 2.12 Biofilm inhibition assay

As previously described, we investigated the inhibitory effects of 31TSP and antibiotics on biofilm, with slight modifications (Chen et al., 2022). The host bacterium Ap31 was cultured to the logarithmic phase, treated with 0.1M PBS (control group), 31TSP (100 μg/mL), ampicillin (16 μg/mL, MIC), or their combination (31TSP + ampicillin) at a final volume of 100 μL/well in a 96-well plate, and incubated at 37°C for 3h, 6h, 9h, 12h, and 24h, respectively. After incubation, all culture media were removed, and the wells were washed three times with 100 μL of 0.1M PBS. The plate was air-dried at room temperature, stained with 100 μL of 0.1% (w/v) crystal violet for 10 min, and then destained with 100 μL of 95% ethanol for 10 min. The remaining biofilm biomass was quantitatively measured at 570 nm wavelength using a microplate reader (BioTek). The anti-biofilm activity was assessed by counting planktonic live bacteria (Wilson et al., 2017). All experiments were independently conducted three times.

### 2.13 Biofilm removal assay

The host bacterium Ap31 was cultured to the logarithmic phase and dispensed into a 96-well plate at 100 μL/well. Based on the growth matrix curve of the host bacterium (**Supplementary Figure 1**), the maximum biofilm biomass formation was determined at 9h, and the plate was incubated at 37°C for 9h. The biofilm was washed twice with 0.1M PBS and then treated with 0.1M PBS (control), 31TSP (100 μg/mL), ampicillin (16 μg/mL, MIC), or their combination (31TSP + ampicillin) at a final volume of 100 μL/well, followed by incubation at 37°C for 3h. At the end of the incubation period, quantification of the remaining biomass was performed using crystal violet as described in the inhibition assay (Chen et al., 2022). Additionally, the anti-biofilm activity was assessed by counting live bacteria within the biofilm (Wilson et al., 2017). After treating the biofilm as described above, wells were washed three times with 0.1M PBS. Subsequently, a pipette device was used to thoroughly mix wells containing the biofilm, converting biofilm cells into planktonic cells. Each sample was serially diluted and subjected to live bacterial counting. All experiments were independently conducted three times.

### 2.14 Scanning electron microscopy observation

Detection of the Impact of 31TSP and Antibiotics on Biofilm Formation and Eradication in *A. pittii*: (1) Biofilm Inhibition Observation: High-pressure sterilized cover slips were placed in a 24-well plate. The host bacterium at the logarithmic phase was treated with 0.1M PBS (control), 31TSP (100 μg/mL), ampicillin (16 μg/mL, MIC), or their combination (31TSP + ampicillin) at a final volume of 1 mL/well. The mixtures were cultured at 37 °C until 9h (maximum biofilm biomass formation). After incubation, all mixtures were removed, and the coverslips were washed three times with 1 mL of 0.1M PBS. Samples treated with depolymerase for varying durations were arranged in reverse chronological order (longest to shortest treatment time) according to the scheduled electron microscopy time slot. The coverlips from all treatment groups were harvested simultaneously and processed for electron microscopy observation. The coverslips were air-dried at room temperature and subjected to gold coating. The impact of 31TSP and antibiotics on biofilm formation in *A. pittii* was observed using a scanning electron microscope (JSM-7900F). (2) Biofilm Eradication Observation: High-pressure sterilized coverslips were placed in a 24-well plate. The host bacterium at the logarithmic phase was dispensed into each well at 1 mL/well and cultured at 37 °C until 9h. After washing twice with 0.1M PBS, the coverslips were treated with 0.1M PBS (control), 31TSP (100 μg/mL), ampicillin (16 μg/mL, MIC), or their combination (31TSP + ampicillin) at a final volume of 1 mL/well. The mixtures were incubated at 37 °C for 3h. After incubation, all mixtures were removed, and the coverslips were washed three times with 1 mL of 0.1M PBS. The coverslips were air-dried at room temperature and subjected to gold coating. The impact of 31TSP and antibiotics on biofilm eradication in *A. pittii* was observed using a scanning electron microscope (JSM-7900F).

### 2.15 Statistical analysis

All data represent the average of three independent experiments, and the results are expressed as the mean ± standard deviation. Statistical significance was analyzed using SPSS Statistics 25.0 software, with *p* < 0.05 considered statistically significant. Graphs were created using Prism 7.

## 3 Results

### 3.1 Morphology and one-step growth curve of phage

**Figure 1:**
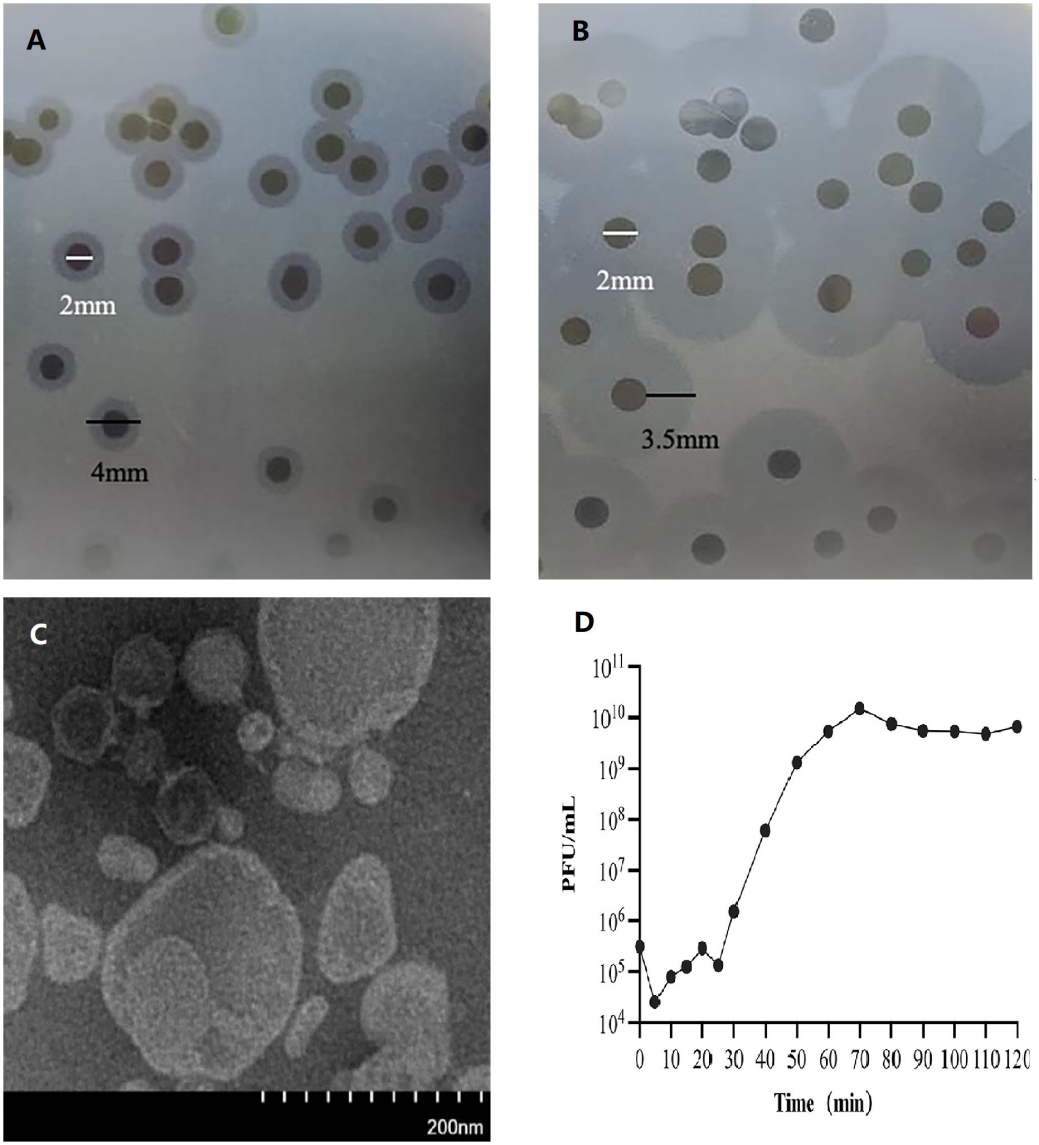
Transparent plaques and halos produced by bacteriophage 31Y. **(A)** Transparent plaques and halos were observed after 7 hours of cultivation. **(B)**Transparent plaques and halos were observed after 20 hours of cultivation. A semi-transparent halo expanded with prolonged incubation time. **(C)** Transmission electron micrograph of bacteriophage 31Y. **(D)** One-step growth curve of bacteriophage 31Y.

When the isolated was inoculated onto a double-layer agar plate containing the host bacterium Ap31 (antibiotic resistance spectrum detailed in **Supplementary Table 1**), transparent plaques surrounded by a semi-transparent halo were formed (diameter approximately 2.5 mm) (**Figures 1A, B**). As the cultivation time increased, the halo area gradually enlarged, with the diameter expanding from approximately 4 mm (cultivation for 7 hours) to about 9 mm (cultivation for 20 hours). Transmission electron microscopy images revealed that the bacteriophage 31Y had an icosahedral head (diameter of about 60 nm) and a non-contractile short tail (about 15 nm) (1C).

After propagation, concentration, and purification, the final titter of bacteriophage 31Y was determined to be 2 × 10^10^ PFU/mL. Infection of the Ap31 strain with bacteriophage 31Y at a multiplicity of infection (MOI) of 0.001 was conducted for a one-step growth curve analysis. As shown in **Figure 1D**, the latent period of bacteriophage 31Y was approximately 25 minutes, the burst period was 45 minutes, and it reached stability after 70 minutes. The burst size of bacteriophage 31Y was approximately 48 PFU/cell.

### 3.2 Whole genome sequencing analysis and structural analysis of depolymerase

The whole genome sequencing results revealed that the size of bacteriophage 31Y genome was 41, 254 bp, arranged as a linear double-stranded genome with a G+C content of 39.26%. The nucleotide composition was 31.15% A, 17.13% C, 22.14% G, and 29.58% T. The annotated genomic sequence reregistration number for bacteriophage 31Y is currently pending and will be provided upon final registration, the sequence is available as supplementary data. Gene prediction using the GeneMarkS tool identified 49 open reading frames (ORFs) within the bacteriophage 31Y genome, covering 37,992 bp with an average length of 775.35 bp. The accession number of bacteriophage 31Y genome in NCBI is PV467367 (*Acinetobacter* phage A31Y, complete genome - Nucleotide - NCBI). According to BlastN analysis and the document of International Committee on Taxonomy of Viruses, Acinetobacter phage A31Y belongs to *Autographiviriles, Autoscriptoviridae*.

**Figure 2:**
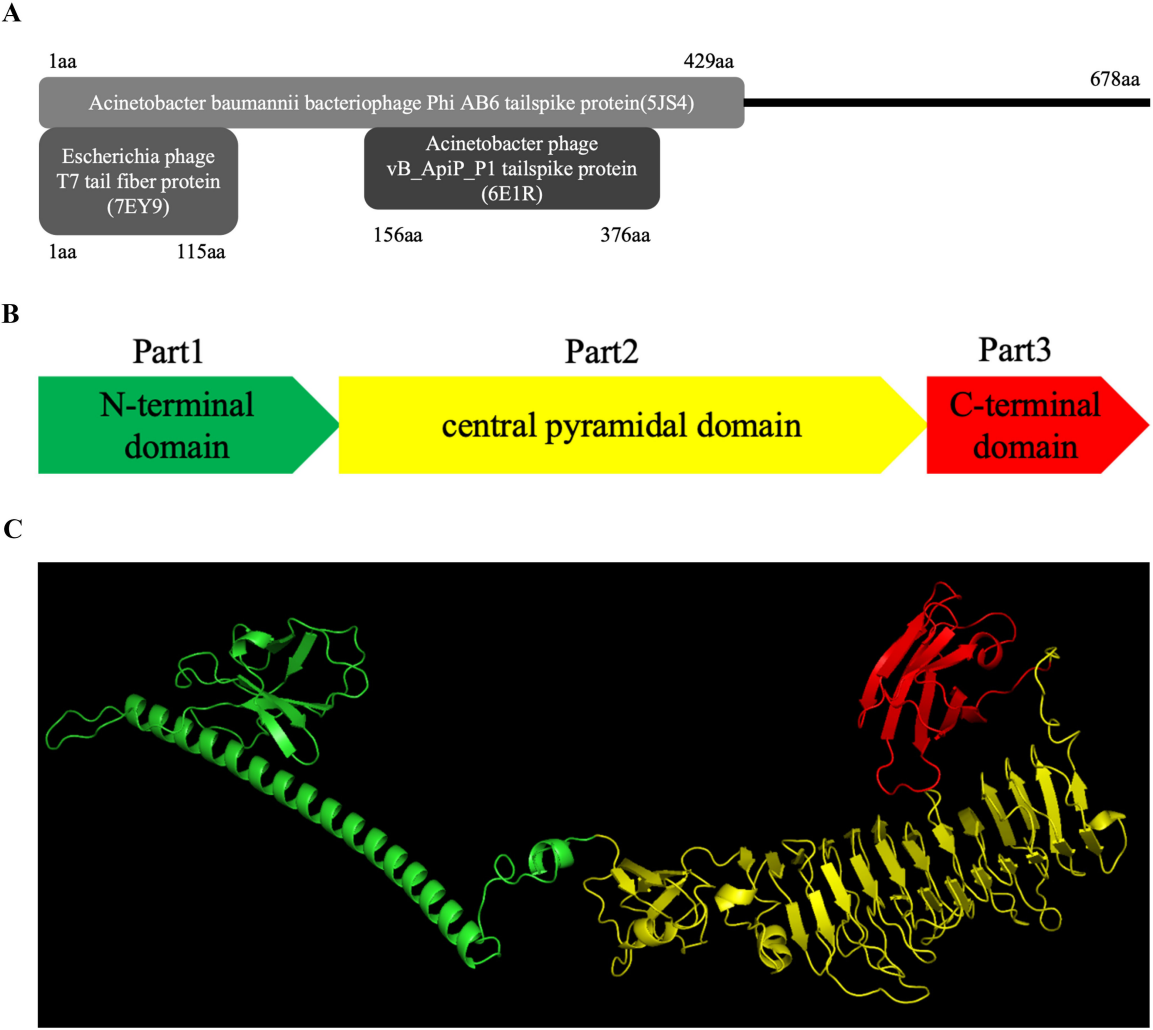
Structural Analysis of 31TSP. **(A)** HHpred analysis identified structural similarity between 31TSP and proteins in the Protein Data Bank (PDB). **(B)** Schematic representation of the structure of 31TSP. **(C)** The predicted spatial structure of monomer 31TSP by AlphaFold 2. The N-terminal domain is represented in green, the central cone-shaped domain in yellow, and the C-terminal domain in red.

Among these ORFs, ORF43 with a size of 678 amino acids, was identified as the gene encoding the tail spike protein as it showed high homology with tail spike proteins of several Acinetobacter phages, such as *Acinetobacter* phages Paty (QQM15083), *Acinetobacter* phage vB_AbaP_ABW311 (CAL1776920) and *Acinetobacter* phage Pipo (QQO92973). These proteins are classified as structural depolymerases originating from these bacteriophages. Although no conserved domains could be found by NCBI Conserved Domain Search, HHpred analysis indicated high structural similarity between the N-terminal region of 31TSP to the N-terminal region of tail fiber/spike protein of the T7 phage (Garcia-Doval and van Raaij, 2012) and *A. baumannii* bacteriophage AB6 (**Figure 2A**).

The predicted structure of the monomer of the depolymerase encoded by bacteriophage 31Y, as determined by AlphaFold 2, revealed three distinct domains (**Figures 2B, C**). The first part exhibited an N-terminal domain with an antiparallel β-sheet structure followed by α-helices, responsible for the attachment of these proteins to the bacteriophage particles, playing a role in the initial interaction with the bacterial host (Drobiazko et al., 2022). The second part constituted a cone-shaped interlocking pyramid domain mainly composed of parallel short β structures, commonly involved in host recognition and exhibiting depolymerase activity (Garcia-Doval and van Raaij, 2012). The simulated third part represented the C-terminal domain, consisting of an antiparallel β-sheet region composed of approximately 100 amino acids.

### 3.3 Expression, purification, and identification of depolymerase

**Figure 3:**
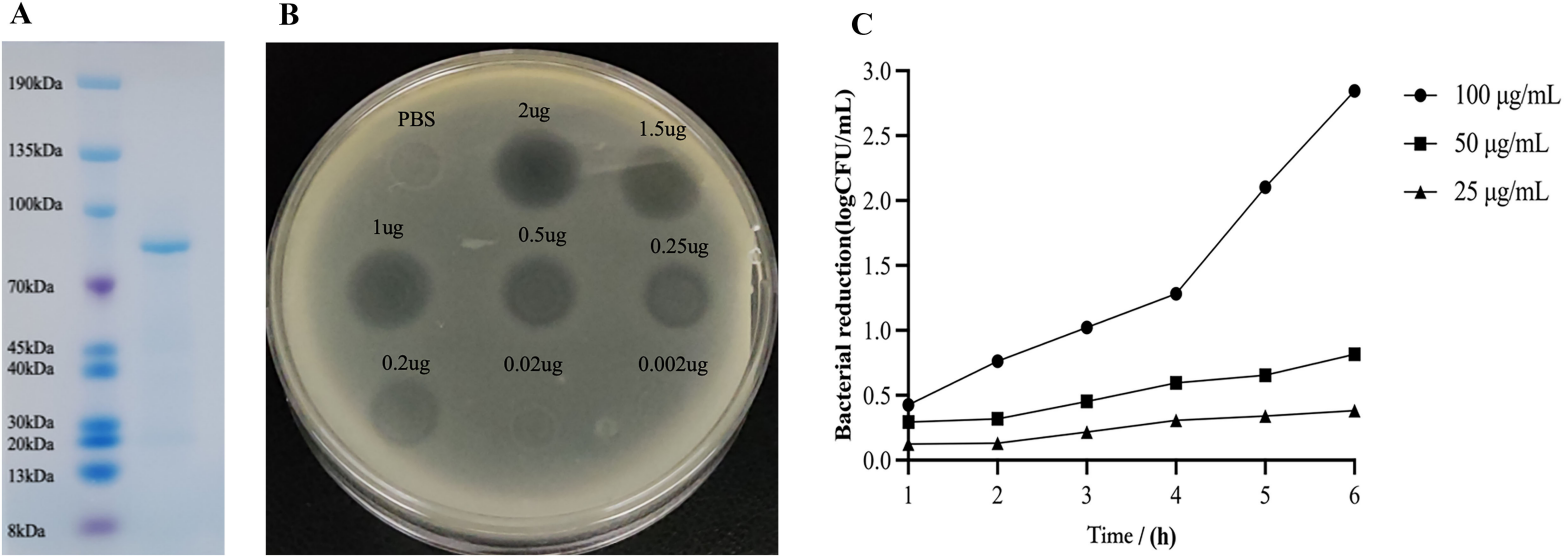
Identification and characterization of 31TSP depolymerase. **(A)** Analysis of purified 31TSP and molecular weight standards by SDS-PAGE. **(B)** Spot assay of Ap31 strain with varying final doses of 31TSP (0.002 ~ 2 μg). **(C)** Living bacterial counts after 31TSP treatment in different concentrations.

The gene ORF43, encoding the tail spike protein, was cloned into an expression vector and purified through Ni-NTA affinity chromatography, yielding a recombinant product at 223 µg/mL concentration. SDS-PAGE analysis confirmed the presence of a recombinant protein, 31TSP, with an approximate molecular weight of 75 kDa (**Figure 3A**). As the total number of amino acids in the protein sequence was 678, the purified protein band observed on the gel corresponds to a depolymerase monomer. To validate the enzyme’s activity post-purification, a plaque assay was conducted. For every test, 5 μL depolymerase in different concentration were dropped on bacteria lawn. The results demonstrated that purified 31TSP formed a semi-transparent halo on the lawn of host strain Ap31 (**Figure 3B**). The area of the semi-transparent halo decreased with decreasing concentrations of 31TSP, disappearing when the total amount of the enzyme spotted onto the bacterial lawn was reduced to 0.02 µg (4 µg/mL). Antibacterial activity experiments indicated a proportional relationship between 31TSP concentration and antibacterial efficacy. The most significant reduction in bacterial count was observed at a concentration of 100 μg/mL (**Figure 3C**). Thus, 31TSP exhibited robust depolymerase activity. However, the group treated with imidazole at the same concentration as the experimental groups showed no antibacterial effect. Please refer to **Supplementary Figure 1** for the results.

### 3.4 Host range of bacteriophage and depolymerase

The depolymerizing capability of 31TSP and the host range of bacteriophage 31Y were assessed using a collection of 98 strains of *Acinetobacter* stored in our laboratory (**Supplementary Table 2**). Overall, among these *Acinetobacter* strains 38 (38/98, 38.8%) were susceptible to depolymerization by 31TSP, while only 5 (5/98, 5.1%) strains were susceptible to lysis by bacteriophage 31Y. Notably, all 5 strains susceptible to bacteriophage 31Y belonged to *A. pittii*. In contrast, among the 38 strains susceptible to 31TSP depolymerization, aside from the 5 A*. pittii* strains lysed by bacteriophage 31Y, an additional 1 A*. pittii*, 1 A*. nosocomialis*, and 31 A*. baumannii* strains were susceptible, significantly having a broader host range than bacteriophage 31Y.

### 3.5 Quantification of depolymerase activity

**Figure 4:**
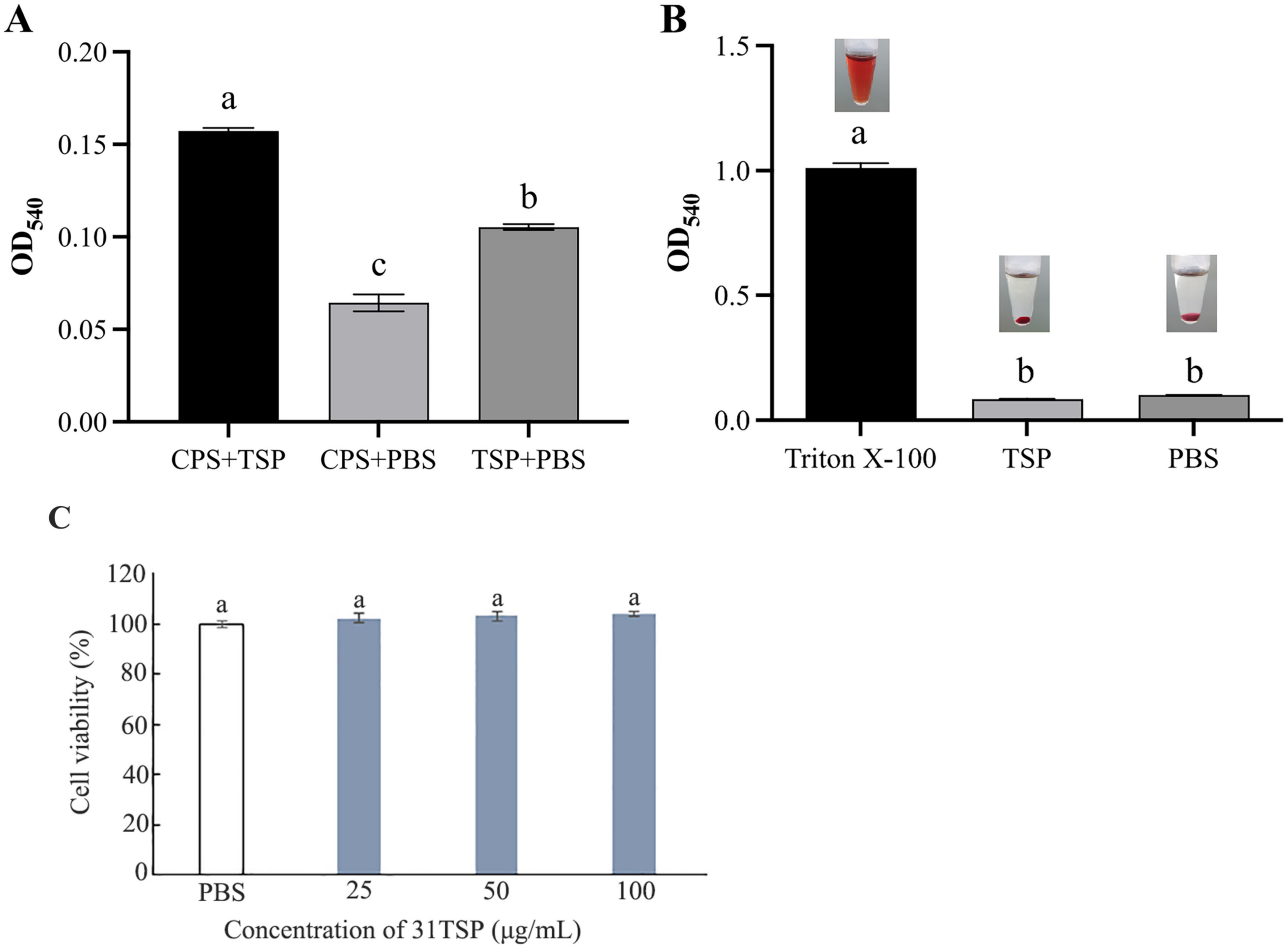
Activity and Safety of 31TSP. **(A)** Quantification of 31TSP activity. Bacterial CPS was co-incubated with 31TSP at 37°C for 1h, with PBS used as controls replacing CPS or 31TSP. The amount of released reducing sugars from EPS was quantified optical density at 540 nm through a reaction with DNS. **(B)** Hemolysis assay of 31TSP. PBS and 0.1% Triton X-100 served as negative and positive controls, respectively. The optical density at 540 nm is presented as mean ± SD (n = 3). **(C)** Cytotoxicity of 31TSP against human embryonic kidney cells (HEK293). Statistical analysis was performed using one-way ANOVA. Groups sharing the same letter are not significantly different, while groups with different letters are significantly different (p < 0.05).

The activity of 31TSP was quantified by measuring the release of reducing sugars from bacterial surface polysaccharides. The OD_540_ values for CPS and 31TSP in PBS were 0.064 ± 0.005 and 0.105 ± 0.002, respectively. In contrast, the OD_540_ for CPS treated with 31TSP was 0.157 ± 0.002 (**Figure 4A**). The increase in OD_540_ for 31TSP-treated CPS indicates the production of reducing sugars, suggesting that surface polysaccharides extracted from the host bacteria were degraded by the depolymerase.

### 3.6 Hemolysis assay and cytotoxicity analysis

Human blood cells and human embryonic kidney cells (HEK293) were used to evaluate the toxicity of 31TSP to mammalian cells. The results of the hemolysis assay (**Figure 4B**) showed no significant difference between 31TSP and the negative control group (PBS group) after 1 hour of incubation with red blood cells, and no hemolysis was observed. **Figure 4C** showed 31TSP has no cytotoxicity against HEK293. These results suggest 31TSP may be a safe therapeutic approach.

### 3.7 Stability of depolymerase

**Figure 5:**
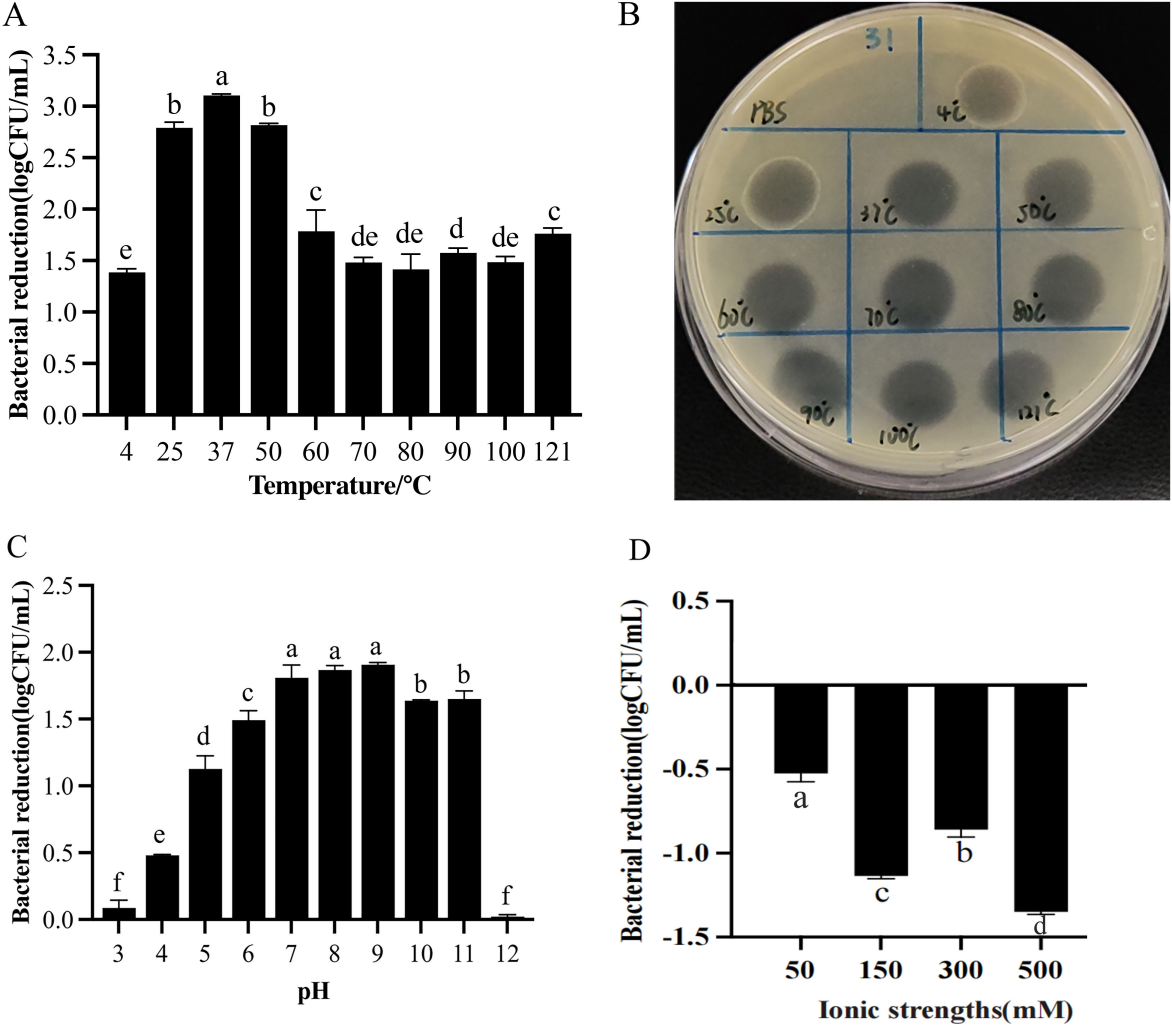
Stability of 31TSP. **(A)** Effect of temperature on 31TSP activity. **(B)** Spot test of 31TSP against Ap31 strain under different temperature treatment conditions. **(C)** Effect of pH on 31TSP activity. **(D)** Effect of ion concentration on 31TSP activity. The values are presented as the log reduction in bacterial count (means ± SD; n = 3), and the statistical analysis was assayed by one-way ANOVA. Groups sharing the same letter are not significantly different, while groups with different letters are significantly different (p < 0.05).

The activity of 31TSP was assessed under various environmental conditions, including temperature, pH, and ion strength (**Figure 5**). Encouragingly, the enzyme demonstrated remarkable temperature stability, maintaining high activity after 30 minutes of treatment across a range of 4 °C to 121 °C (**Figures 5A, B**). The maximum reduction in viable bacterial count occurred at 37 °C, reaching 3.104 ± 0.016 log, indicating the highest activity. Even after treatment at 100 °C and 121 °C for 30 minutes, the enzyme still caused reductions of 1.484 ± 0.055 and 1.762 ± 0.053 log, respectively. Furthermore, the enzyme remained active over a broad pH range from 5 to 11 (**Figure 5C**), and even at pH 4 some depolymerase keep weak activity.

Ion concentration significantly decreased the activity of 31TSP, with a notable impact even at 50 mM ion concentration, resulting in a reduction of 0.525 ± 0.050 log compared to the control group (**Figure 5D**). The influence of ion concentration on depolymerase activity appeared unrelated to a dose-dependent effect.

### 3.8 The effect of depolymerase on serum bactericidal activity

**Figure 6:**
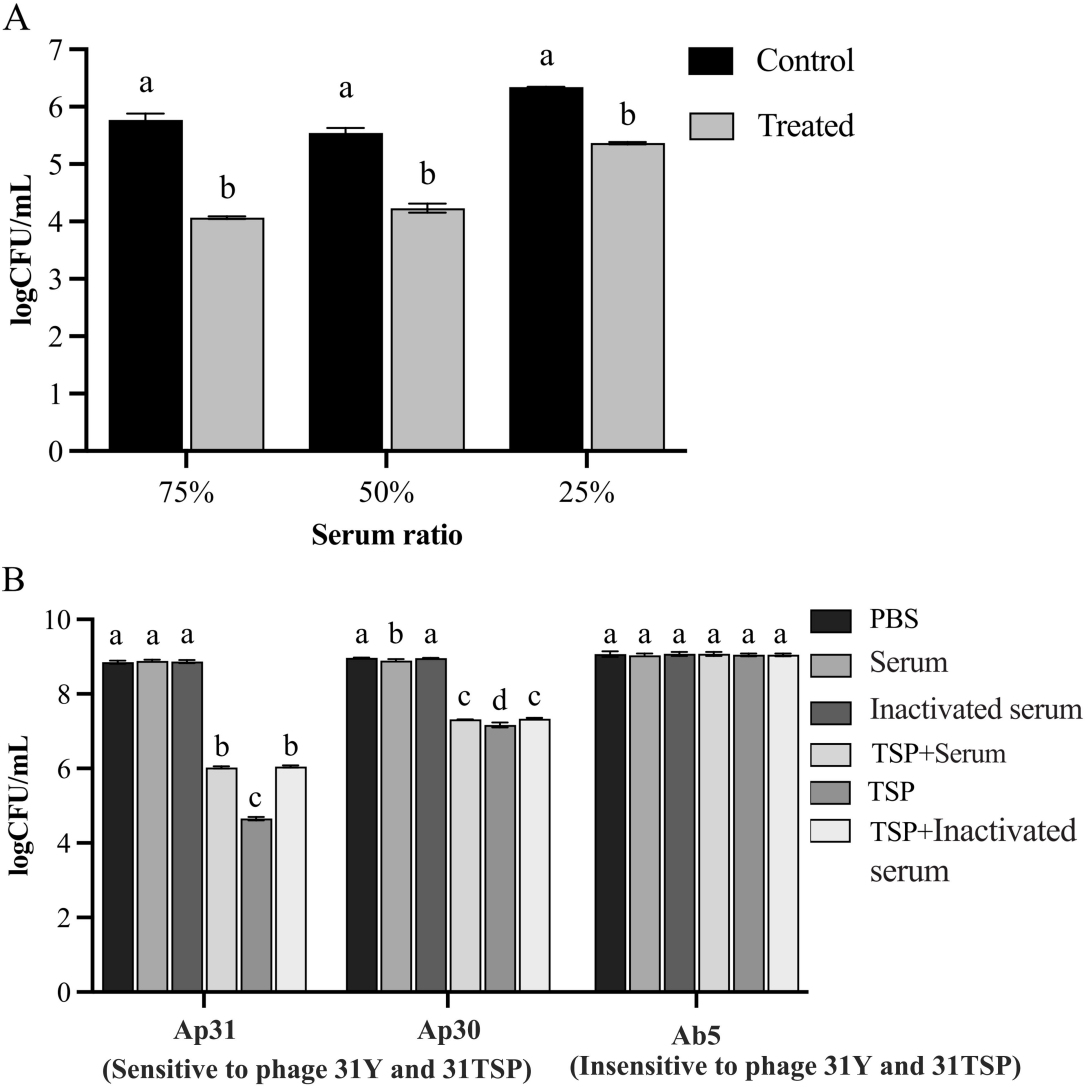
Serum killing experiments. **(A)** Determination of the optimal volume content of 31TSP-treated bacteria to serum. 31TSP pre-treated Ap31 was incubated with human serum at a volume ratio of 3:1, 1:1 or 1:3 for 3 hours and viable bacteria were counted. **(B)** *A. pittii* Ap30 and *A. pitti* Ap31are sensitive to 31TSP and bacteriophage 31Y, *A. baumannii* Ab5 was insensitive to 31TSP and bacteriophage 31Y. These strains were used as target bacteria to test the combination of 31TSP with serum or inactivated serum. 31TSP-treated bacteria were incubated with serum or inactivated serum at a 3:1 volume content. Then the living bacteria were cultured and counted. Data is expressed as mean ± SD (n = 3), and statistical analysis was performed by one-way ANOVA. Groups sharing the same letter are not significantly different, while groups with different letters are significantly different (p < 0.05).

Compared to 31TSP untreated bacteria, the number of host bacteria (Ap31) treated with 31TSP decreased when mixed with varying volumes of serum (*P* < 0.05), but the differences were not observed among different serum proportion groups (**Figure 6A**). In subsequent experiments, a serum proportion of 25% was used.

To assess the role of serum, we chose *A. pittii* Ap30, Ap31 and *A. baumannii* Ab5, as the target bacteria. Ap31 and Ap30 are sensitive to phage 31Y and 31TSP, but Ab5, is insensitive to phage 31Y and 31TSP. These three test strains exhibited resistance to serum killing, continuing to grow in human serum in the absence of 31TSP. Conversely, after treatment with 31TSP and subsequent incubation with active serum or inactive serum for 3 hours, the bacterial count of host strain Ap31 and Ap30 are higher than TSP treated groups (*P* < 0.05). While the insensitive strain Ab5, all experimental groups exhibited almost no reduction in bacterial count than PBS control (**Figure 6B**). Therefore, the experimental *Acinetobacter* strains are resistant to serum killing, and serum and inactivated serum showed inhibitory effect on 31TSP.

### 3.9 Anti-biofilm activity

**Figure 7:**
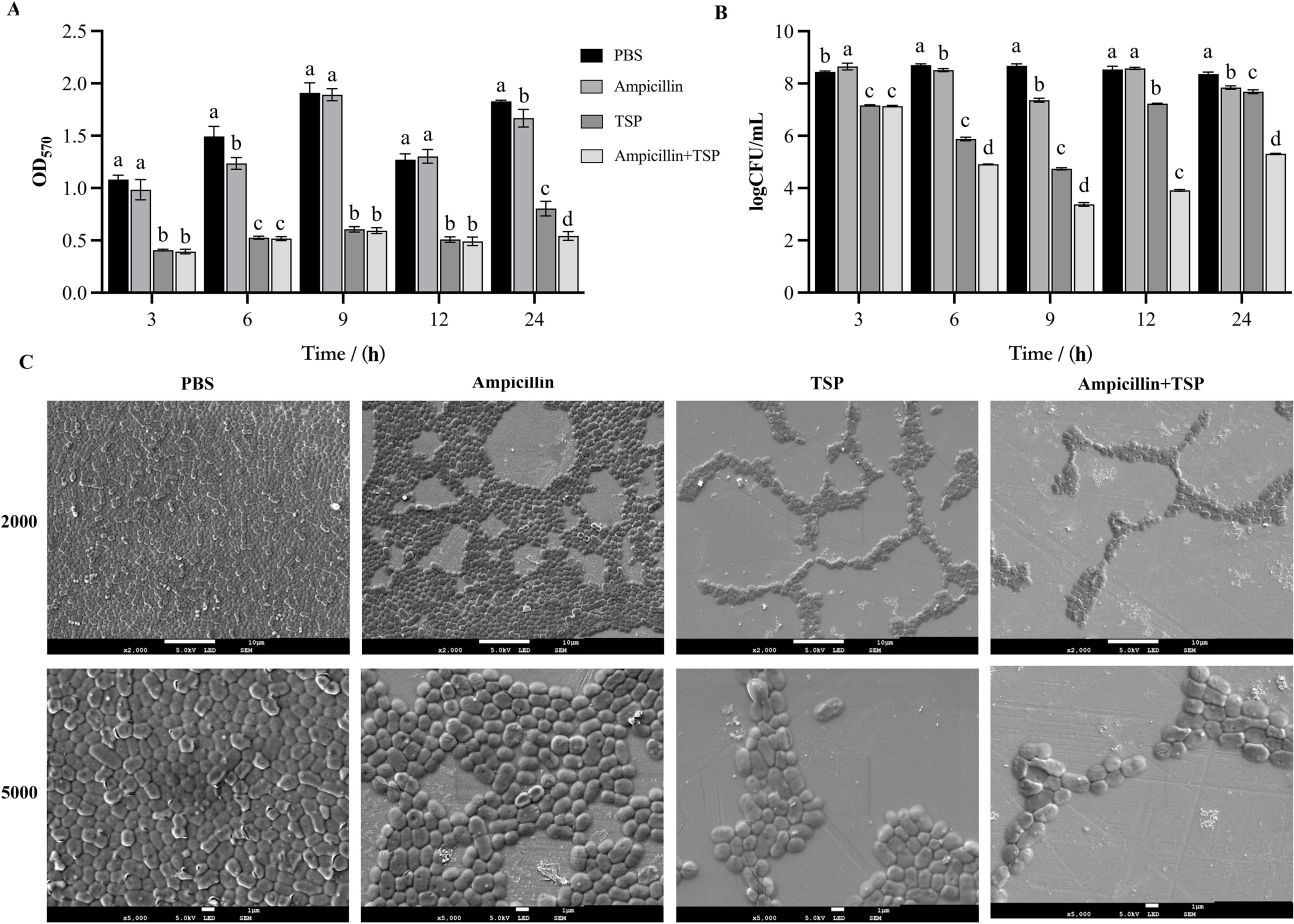
Inhibition of Biofilm Formation by 31TSP and Ampicillin. **(A)** Crystal violet staining of biofilms. **(B)** Living planktonic bacterial counting assay. **(C)** Scanning electron microscopy observation at 24 hours. Ap31 bacterial cultures were treated with PBS (control group), 31TSP (100 μg/mL), ampicillin (16 μg/mL, MIC), or their combination (31TSP + ampicillin), followed by crystal violet staining, planktonic bacterial counting, and scanning electron microscopy observation. Data are presented as mean ± SD (n = 3), and statistical analysis was conducted using one-way ANOVA. Different letters (a-c) indicate significant differences (p < 0.05).

We first evaluated the inhibitory effects of 31TSP on biofilm formation using crystal violet staining. Quantitative analysis demonstrated that while ampicillin alone (16 μg/mL, MIC) showed partial inhibition of biofilm formation compared to PBS controls at both 6 and 24 hours (p < 0.05), 31TSP alone exhibited significantly stronger inhibitory effects (p < 0.05). The combination of 31TSP and ampicillin showed the most pronounced biofilm inhibition, particularly at the 24-hour time point (**Figure 7A**). However, at earlier points (6 hours), no significant difference in biofilm inhibition was observed between 31TSP alone and the combination treatment (p > 0.05).

Viable cell counting results demonstrated that, with prolonged incubation time, the planktonic bacterial count in the PBS control group tended to stabilize. In comparison to the PBS control group, the exclusive use of 31TSP resulted in a reduction of planktonic bacterial counts by 1.280, 2.825, 3.940, 1.306, and 0.675 log at 3, 6, 9, 12, and 24 hours, respectively. Furthermore, the combination with ampicillin further enhanced this inhibitory effect, leading to reductions of 0.027, 0.964, 1.360, 3.319, and 2.379 log at the respective time points (**Figure 7B**). Although the inhibitory effect of sole 31TSP application on planktonic bacterial growth diminished significantly at 12 and 24 hours, the combined treatment with ampicillin effectively suppressed planktonic bacterial growth. SEM further validated the results (**Figure 7C**). Our findings unequivocally confirm the efficacy of both sole 31TSP application and its combination with ampicillin in preventing *A. pittii* biofilm formation.

**Figure 8:**
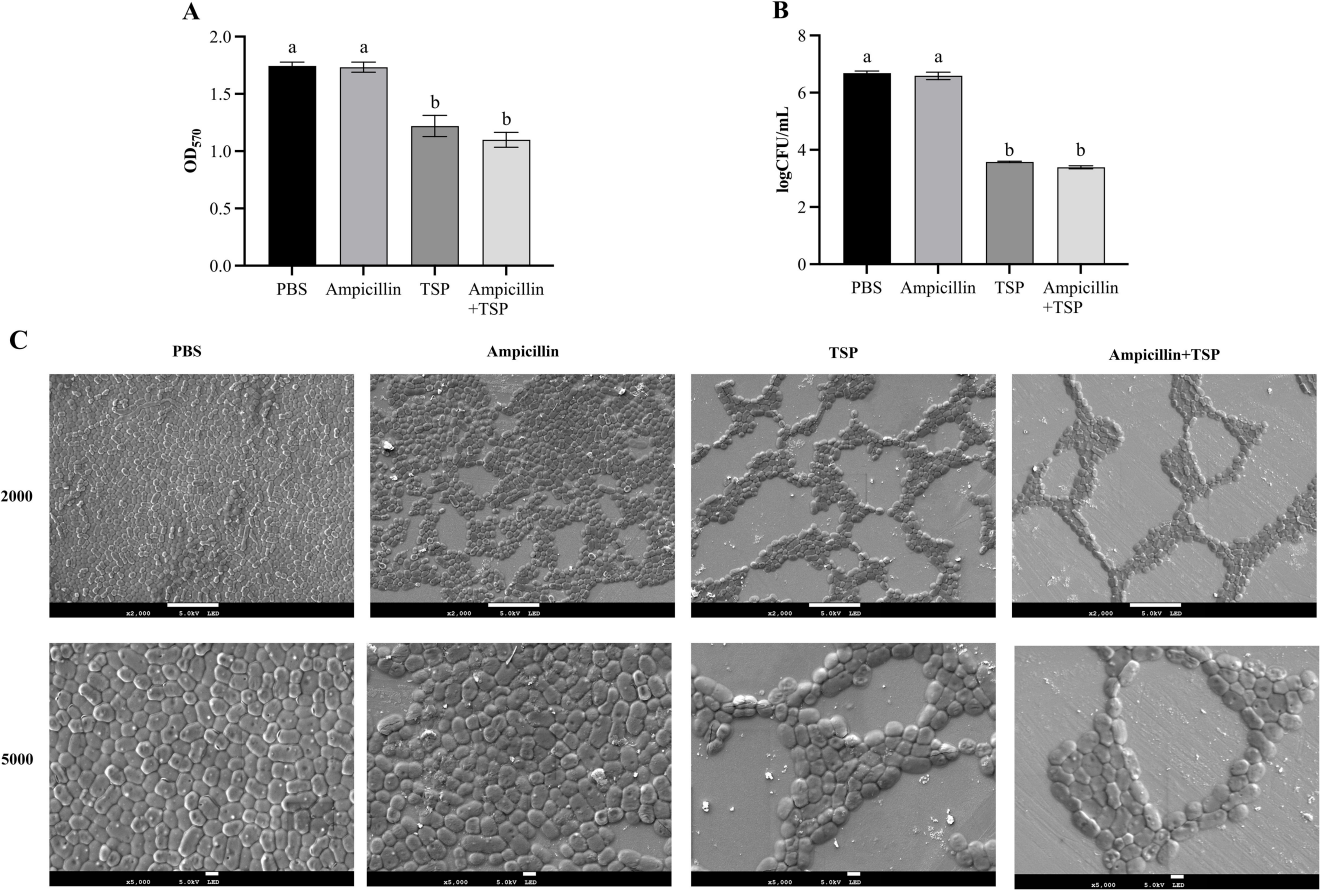
Disruption of Pre-formed Biofilms by 31TSP and Ampicillin. **(A)** Crystal violet staining. **(B)** Ap31 counting assay. **(C)** Scanning electron microscopy observation. Ap31 biofilms were initially cultured for 9 hours, followed by treatment with PBS (control), 31TSP (100 μg/mL), ampicillin (16 μg/mL, MIC), or their combination (31TSP + ampicillin) for 3 hours. Subsequently, crystal violet staining, bacterial counting, and scanning electron microscopy observation were performed. Data are presented as mean ± SD (n = 3), and statistical analysis was conducted using one-way ANOVA. Groups sharing the same letter are not significantly different, while groups with different letters are significantly different (p < 0.05).

Subsequently, we assessed whether 31TSP alone or in combination with ampicillin could disrupt pre-formed biofilms. Crystal violet staining results indicated that, compared to the PBS control group, the sole administration of ampicillin failed to disrupt pre-formed biofilms. In contrast, the exclusive use of 31TSP and its combination with ampicillin effectively disrupted the pre-formed biofilms (p < 0.05). However, there were no significant differences between the sole use of 31TSP and its combination with ampicillin (p > 0.05) (**Figure 8A**).

Based on viable cell counting results, sole treatment with 31TSP could disrupt the host bacteria, reducing the residual host bacteria by 3.104 log compared to the PBS control group. The combination treatment further decreased the remaining host bacteria by 0.095 log, with no significant differences observed between the sole use of 31TSP and its combination with ampicillin (p > 0.05). In the absence of 31TSP, ampicillin exhibited inefficient killing of bacteria embedded in the biofilm, resulting in a modest reduction of 0.095 log (**Figure 8B**). SEM observations were consistent with the aforementioned results (**Figure 8C**). These data suggest that 31TSP can effectively eradicate pre-formed biofilms and enhance the anti-biofilm activity of ampicillin.

## 4 Discussion

*A. pittii*, as a member of the ACB complex, often faces misidentification due to its phenotypic similarity to *A. baumannii*, exacerbated by technical limitations in clinical laboratory identification methods (Pailhoriès et al., 2018; Molina et al., 2010; Chen et al., 2018). However, with an increasing number of clinical isolates and advancements in molecular identification techniques (Teixeira et al., 2017; Marí-Almirall et al., 2019; La Scola et al., 2006), the relevance of the *A. pittii* species is growing. A research report from a French hospital indicates that *A. pittii* is more frequently isolated from blood cultures compared to *A. baumannii*, underscoring its clinical significance (Pailhoriès et al., 2018). Studies also suggest a 17% mortality rate within 28 days for *A. pittii*-induced bacteremia (Liu et al., 2017). Recent multicenter surveys in Japan reveal *A. pittii* as the most common species causing invasive *Acinetobacter* infections (Kiyasu et al., 2020). Moreover, *in vitro* and *in vivo* models highlight the higher pathogenicity of *A. seifertii* and *A. pittii* compared to *A. baumannii* and *A. nosocomialis* (Cosgaya et al., 2019; Kuo et al., 2013). With increasing attention on *A. pittii*, cases of *A. pittii* carrying carbapenemase NDM-1 have been frequently reported, contributing to increased carbapenem resistance and alterations in resistance mechanisms (Bogaerts et al., 2013; Chen et al., 2019; Pailhoriès et al., 2017). *A* recent study reported a severe pneumonia case associated with a pan-drug-resistant *A. pittii* infection in a patient with chronic obstructive pulmonary disease three years post-double lung transplant (Yang et al., 2021).

In response to the growing resistance of *A. pittii*, this study investigates the potential of bacteriophage-encoded depolymerases as a novel therapeutic strategy. Understanding of the pathogenic mechanisms of *A. pittii* is currently limited. However, it is known that *A. pittii* possesses a thick capsular polysaccharide (CPS) layer surrounding its cells, considered a crucial virulence factor, hindering the penetration of certain peptide antibiotics and shielding the bacterium from host immune system attacks (Drobiazko et al., 2022). Importantly, bacteriophage-encoded depolymerases have the capability to degrade bacterial CPS, rendering the bacteria more susceptible to host immune system mediated killing (Chen et al., 2022; Liu et al., 2020; Majkowska-Skrobek et al., 2018).

According to HHpred analysis, the depolymerase 31TSP exhibits structural similarity to tail spikes from various bacteriophages. Its N-terminal portion shares a structural resemblance with the N-terminal segment of the T7 bacteriophage tail fiber protein, participating in the initial steps of bacteriophage interaction with the bacterial host (Hsieh et al., 2017; Lin et al., 2017). Predictions of the TSP domain structure by AlphaFold 2 reveal three regions: N-terminal, central pyramid, and C-terminal, consistent with recently reported depolymerase structures derived from *A. baumannii* bacteriophages Drobiazko et al., 2022). After the expression and purification of 31TSP, it was discovered that the host range of depolymerase is wider than that of phage 31Y. Sensitive host of phage 31Y is limited to five strains of *A. pittii*, but sensitive host of 31TSP includes seven strains of *A. pittii*, one strain of *A. nosocomialis*, and 31 strains of *A. baumannii*. We also tested the sensitivity of some Gram-negative bacilli, such as *Pseudomonas aeruginosa*, *Klebsiellas pneumoniae*, *E. coli* and *Enterobacter hormaechei*, but no sensitive strains were found. As 31TSP showed a wider host range than original phage, methods on the T7 phage research could be used to investigate the mechanisms underlying host range alteration in future studies (Avramucz et al., 2021., Holtzman et al., 2024).

According previous notion (Chen et al., 2022; Liu et al., 2019a; Lin et al., 2017), the depolymerases generally do not directly kill bacteria during antibacterial therapy. But in this study, according to viable cell counting on bacteria treated with 31TSP, it was found that the number of bacteria in the depolymerase-treated group was significantly lower than that in the untreated group (**Figure 3C**). Furthermore, it has been demonstrated in many phage depolymerases exert their antibacterial effects in a trimeric form. For example, in *Acinetobacter*, the depolymerase Dpo71 from an *A. baumannii* phage exerts its antibacterial activity as a trimer (Abdelkader et al., 2022). However, in recent years, studies have also confirmed that some depolymerases exert their antibacterial function independently of trimer formation, such as the depolymerase from *Klebsiella pneumoniae* phage KP34 and the depolymerase that degrades the K2 serotype capsular polysaccharide of *K. pneumoniae* (Maciejewska et al., 2023; Ye et al., 2024). In this study, the structure of 31TSP was not verified by size exclusion chromatography or circular dichroism analysis as in research of Dpo71. However, when we subjected the nickel-column purified depolymerase to low-temperature ultrafiltration using filters of different molecular weight cut-offs (MWCOs), we found that not only the flow-through fraction from the >100 kDa filter retained the ability to inhibit the growth of susceptible bacteria, but the <100 kDa fraction also contained material capable of inhibiting susceptible bacterial growth. Notably, the monomeric molecular weight of 31TSP is approximately 70 kDa. Therefore, further investigation is warranted regarding whether 31TSP’s antibacterial function is dependent on trimer formation.

31TSP demonstrates remarkable stability in a wide range of physicochemical conditions, maintaining its activity between temperatures of 4°C to 121°C and within a pH range of 5-11. This impressive tolerance to extreme conditions suggests a correlation with the structural properties of bacteriophage depolymerases. These enzymes have evolved to endure harsh external environments during the course of evolution, ensuring the infectivity of bacteriophages (Oliveira et al., 2019). The observed heat resistance of 31TSP, even after exposure to high-pressure treatment at 121°C for 30 minutes, appears to be reported for the first time. The stability of 31TSP across such a broad range of temperatures and pH levels makes it particularly promising for use in clinical settings or food industry.

In previous research, depolymerase contributing to enhanced susceptibility of bacteria to serum complement-mediated killing (Liu et al., 2020; Majkowska-Skrobek et al., 2018). However, in our serum bactericidal assay, target bacteria showed resistant to serum killing. In 31TSP sensitive strains, serum or inactivated serum even showed slightly inhibit effects to TSP (**Figure 6**). The reasons for this difference lie in the mechanism of action of TSP, a mechanism that remains elusive to date (Chen et al., 2022). Further studies are needed to elucidate the exact molecular mechanisms behind 31TSP’s activity, as this could provide deeper insights into its clinical potential.

The formation of biofilms is a major factor contributing to the chronicity of bacterial infections and the increased antibiotic resistance (Sharma et al., 2019; Costerton et al., 1999). Depolymerases have proven valuable in inhibiting and/or eradicating biofilms formed by *Klebsiella* (Li et al., 2022; Wu et al., 2019), Shiga toxin-producing *Escherichia coli* (Chen et al., 2020), and *A. baumannii* (Shahed-Al-Mahmud et al., 2021). The persistence and antibiotic resistance of *A. pittii* infections are strongly associated with biofilm formation. 31TSP demonstrates dual antibiofilm activity, exhibiting both preventive effects on biofilm formation and disruptive effects to mature biofilm. These properties suggest its potential as an adjunctive therapeutic agent to enhance the effectiveness of conventional antibiotics against biofilm-associated infections.

In this study, 31TSP alone demonstrated significant anti-biofilm activity, effectively preventing biofilm formation and disrupting mature biofilms. We also tested the combination of 31TSP and ampicillin, which showed enhanced biofilm inhibition during prolonged treatment (24 h), but no significant improvement over 31TSP monotherapy was observed in disrupting pre-formed biofilms. As the ampicillin is not the first choice for treatment for clinical *Acinetobacter* infections, the combination test should choose carbapenem as test antibiotics, but all strains sensitive to 31TSP in our lab are also sensitive to carbapenem. Notably, beyond its activity against *A. pittii*, 31TSP demonstrated cross-species inhibitory effects against clinically isolated *A. nosocomialis* and *A. baumannii* strains. This broad host range not only enhances its potential clinical applicability but also provides valuable insights for future investigations into its host recognition mechanisms.

## 5 Conclusion

The depolymerase 31TSP demonstrates a broad host range to *A. pittii*, *A. baumannii*, and *A. nosocomialis*, maintaining functional stability across extensive pH (5–11) and temperature (4–121 °C) ranges. As a safe and effective antimicrobial agent, 31TSP exhibits dual functionality: (1) enzymatic degradation of bacterial capsular polysaccharides (CPS) and (2) direct bactericidal activity. While 31TSP alone significantly inhibits biofilm formation and disrupts mature biofilms (p < 0.05), its combination with ampicillin shows synergistic enhancement in biofilm prevention during prolonged exposure (24 h). These findings position phage-derived depolymerases, particularly 31TSP, as promising therapeutic candidates for combating *Acinetobacter* infections, especially in biofilm-associated cases.

## Data Availability

The datasets presented in this study can be found in online repositories. The names of the repository/repositories and accession number(s) can be found in the article/**Supplementary Material**.
