# Correction: Identification and functional characterization of a novel *Acinetobacter pittii* bacteriophage-encoded depolymerase

**DOI:** 10.3389/fcimb.2025.1722705

**Published:** 2025-10-31

**Authors:** Na Zhang, Wei Li, Xue Du, Danish Daniyal, Meng-ai Feng, Jiaoyang Xu, Ziqin Yang, Hailin Jiang, Muhammad Sheraz, Honglan Huang, Santasree Banerjee, Hongyan Shi

**Affiliations:** ^1^ Department of Pathogen Biology, College of Basic Medical Sciences, Jilin University, Changchun, Jilin, China; ^2^ Department of Genetics, Qujing Maternal and Child Health-care Hospital, Qujing, Yunnan, China; ^3^ Department of Clinical Laboratory, China-Japan Union Hospital of Jilin University, Changchun, Jilin, China; ^4^ Clinical Laboratory, Affiliated Hospital of Changchun University of Chinese Medicine, Changchun, China; ^5^ Department of Genetics, College of Basic Medical Sciences, Jilin University, Changchun, China

**Keywords:** *Acinetobacter pittii*, bacteriophage, depolymerase, antibacterial activity, biofilm, antibiotic combination

Affiliation 3 of this article was erroneously written as: “Department of Clinical Laboratory, The Third Bethune Hospital of Jilin University, Changchun, Jilin, China”. It has been corrected to: “Department of Clinical Laboratory, China-Japan Union Hospital of Jilin University, Changchun, Jilin, China”.

The original version of this article has been updated.

