# Supplementary material for: Identification and functional characterization of a novel Acinetobacter pittii bacteriophage-encoded depolymerase
Source: Front Cell Infect Microbiol. 2025 Sep 12;15:1608526. doi: 10.3389/fcimb.2025.1608526 (PMC12463973; doi:10.3389/fcimb.2025.1608526)

Supplementary material

Sequence of A31Y-TSP

ATGAACACACTACGATCATTTACAGAGACTGTAGTGACTACCCCTACAGATACTTTCCCA
ATCAGTTTTGAATTTGATGAGAAGTATGATGTAGTCCATGTCTATGTAGACGGTAAAGTA
GCAGAGGACGAAGGGTACACTGTTACCCTCGTTAACCCTGTTACTTTAAAAGTTGAACCT
GCTATTCCAGAAGGTACGGTTCGTATTGAACGCGAAACAGATATTGATAAGATGAAGTAC
ATCTTTGATGCTGGTGCGTTATTCATTGACCAGAATGTGGATGCAGATTTTAAACAAATC
GTGCACTCCCAGCAAGAAGTGCGTGATGGTTTTATTAAACTACGGGGCGATGTACTACCA
TTAGTATACGGTTTACAAGAGGCTCTGAAACAAGCACAAGAGGCTAGCGAAGCCGCTCAA
GAAGCAGCTGATGCTGCCGAAGAAGCTGCTGCGTTAGTTAGTGGTGCGGTTACGTCTGTC
AAAACTATTGCAGACTTAAAAGCATTACCTACATGGGATGGGCGTGTCGTCGTTATGCAA
GGGCATACTGAATTGGGTAAGAAATCCACTATATTCAGATATGATCCTACTAACACAGAT
ACCCATAACAACTATTCTGTAGTTAAACCTAATACTGGTACAGGTAATTGGTTGGCAGTA
GATACTGCTGTGTACGAAAGTTCTCAGTTAGGGTGTGGTATTGCTATTACTAATAACGAT
GTATTAATTAACCCATTATTAGCTACGTCAGGGTCTATTACAATCCGTGAGGAGTTGCCT
ACTACGAACTCAATTAATGTACAGAGTTACAATGATATTCGTGGTCTAGGTACACGAGCA
GGGTTTAGGTATAATGGGCCGAGTGGTACTGCCGATACTTTAGAAGGTCGCTTACCCGTT
GTTAAACTATTCAAGGATAATGCTCAGGCAGTCAGTCAATCTAGGTTAGAGAACTTAACC
ATCCATGCAAACTACAAACCTTATGTAACTGCTGTAGATGCCCGATACCTTACAGGTCAG
TCTAAACTACGTGAGTTAATCATTCGTGGTGTTACAGATACATCTGTAGGGTTAGTGCTC
AACAAGATGTGGTACGATAAGATCAGTGATGTGTTCATTAGTGGTTATGATCCTAGTAAA
CCTGATGGTTCTCGTTGGGGTACTGGTGTGGTTATTGAATCTAAGTACTCTTCCGATCCT
ACATCACCAGACTACGGGGCACAGATCAATGCTTTGACAGTAGATGTATCATGTCATAGT
TTAAAGGTAGGTTACTTAATTCGACCAAATAACTACATCTACGGTTTAAATATTAATACA
ATGCCTACTGTAGAGAACTGCGACATTGGCTTCAAGATGGAATCTGCACCTGCATCTGTA
GATTATCAAGTACGGCAAAGCTGTATTTCTGCGTACTTTGAAAACAACGGTATTGATATT
CAGTGGGGTACACCTAGTACCACTAAAGCACGTAACTCTAAACACGTATGGTTGAACTGT
TCTTTTGATGATGTTCATTCACGTATTGAGTTATATGAAGGACATCATACCTTCATCGGA
TGTAAGGGTATTAAGACTCTAGTGTGTGGTCAATATGCTCAAGCTGAGCTTATTAACACA
GCACGACCTACAACTATCACTGATGATTACGGTAACGTAACTGTACGTCAAGACCCTATG
TCTCAGATTACATCTGGCTCGTATCAAAATGTACGTACACGTTCTCAAGGTATGTTGAAA
CTCAAGGCTACTGTCAATGCGGGTGCCACTGGTACGTTTGATTTAGGTTCTGTATTAAAT
ACTACTATCGCTTCTGAGGGGCAAACTGGTTTAGTGCGTATCTTATCACGACGCTCTTAT
GATACTGCGCCTGTTATTGCACAAGGTGTTATCTTACGTAAATCTACAGGAACTACGTTT
ACACCTATTGGATCTGTACCTACAGGTATGACTATCAGCATTACAGGTACCGTGTTAAGT
GTATACGAGACTAGAGGTGACACAAAGTTTTTAGATATTATTTTTAATCCAGATTAA

Supplementary Table 1 Antibiotic resistance spectrum of *A. pittii* Ap31

Antibiotics	MIC (ug/mL)	Sensitivity	Antibiotics	MIC (ug/mL)	Sensitivity
Amikacin	<=8	S	Piperacillin	16	S
Gentamycin	8	I	Amoxicillin/Clavulanic acid	>16/8	R
Imipenem	<=1	S	Ampicillin/Sulbactam	<=4/2	S
Meropenem	<=1	S	Piperacillin/Tazobactam	<=4/4	S
Cefazolin	>16	R	Compound sulfanilamide	<=0.5/9.5	S
Ceftazidime	4	S	Chloramphenicol	>16	R
Cefotaxime	8	S	Ciprofloxacin	<=0.5	S
Cefepime	4	S	Levofloxacin	<=1	S
Aztreonam	16	R	Tetracycline	<=2	S
Ampicillin	16	R			

Note: R: Resistant I: intermediate S: sensitive

Supplementary Table 2 The host range of 31Y and 31TSP

Strain number	Bacterial strain	Sensitivity to phage 31Y	Sensitivity to depolymerase 31TSP
1	*A. baumannii* (Ab1)	-	+
2	*A. baumannii* (Ab2)	-	+
3	*A. baumannii* (Ab3)	-	+
4	*A. baumannii* (Ab4)	-	-
5	*A. baumannii* (Ab5)	-	-
6	*A. baumannii* (Ab6)	-	-
7	*A. baumannii* (Ab7)	-	-
8	*A. baumannii* (Ab8)	-	-
9	*A. baumannii* (Ab9)	-	-
10	*A. pittii* (Ap10)	-	+
11	*A. baumannii* (Ab11)	-	+
12	*A. baumannii* (Ab12)	-	-
13	*A. baumannii* (Ab13)	-	-
14	*A. baumannii* (Ab14)	-	-
15	*A. baumannii* (Ab15)	-	-
16	*A. baumannii* (Ab16)	-	+
17	*A. baumannii* (Ab17)	-	+
18	*A. baumannii* (Ab18)	-	+
19	*A. baumannii* (Ab19)	-	-
20	*A. baumannii* (Ab20)	-	-

21	*A. baumannii* (Ab21)	-	+
22	*A. baumannii* (Ab22)	-	-
23	*A. baumannii* (Ab23)	-	-
24	*A. baumannii* (Ab24)	-	-
25	*A. baumannii* (Ab25)	-	-
26	*A. baumannii* (Ab26)	-	-
27	*A. baumannii* (Ab27)	-	+
28	*A. baumannii* (Ab28)	-	+
29	*A. baumannii* (Ab29)	-	-
30	*A. pittii* (Ap30)	+	+
31	*A. pittii* (Ap31)	+	+
32	*A. baumannii* (Ab32)	-	+
33	*A. baumannii* (Ab33)	-	+
34	*A. baumannii* (Ab34)	-	-
35	*A. baumannii* (Ab35)	-	-
36	*A. baumannii* (Ab36)	-	-
37	*A. baumannii* (Ab37)	-	+
38	*A. baumannii* (Ab38)	-	-
39	*A. baumannii* (Ab39)	-	-
40	*A. baumannii* (Ab40)	-	+
41	*A. baumannii* (Ab40)	-	+
42	*A. baumannii* (Ab40)	-	-
43	*A. pittii* (Ap43)	+	+
44	*A. baumannii* (Ab44)	-	-
45	*A. baumannii* (Ab45)	-	-
46	*A. pittii* (Ap46)	+	+
47	*A. baumannii* (Ab46)	-	-
48	*A. baumannii* (Ab47)	-	-
49	*A. baumannii* (Ab48)	-	-
50	*A. nosocomialis*	-	+
51	*A. baumannii* (Ab1)	-	+
52	*A. pittii* (Ap52)	+	+
53	*A. pittii* (Ap53)	-	+
54	*A. baumannii* (Ab54)	-	-
55	*A. baumannii* (Ab18)	-	-

56	*A. baumannii* (Ab65)	-	-
57	*A. baumannii* (Ab40)	-	-
58	*A. baumannii* (Ab140)	-	-
59	*A. baumannii* (Ab4new)	-	-
60	*A. baumannii* (Abry 1)	-	-
61	*A. baumannii* (Ab58)	-	-
62	*A. baumannii* (Ab50)	-	+
63	*A. baumannii* (Ab94)	-	+
64	*A. baumannii* (Abry28)	-	+
65	*A. baumannii* (Abry21)	-	-
66	*A. baumannii* (Ab125)	-	-
67	*A. baumannii* (Abry18)	-	-
68	*A. baumannii* (Ab17new)	-	-
69	*A. baumannii* (Ab77)	-	-
70	*A. baumannii* (Abry17)	-	-
71	*A. baumannii* (Ab23)	-	-
72	*A. baumannii* (Ab42)	-	-
73	*A. baumannii* (Ab136)	-	+
74	*A. baumannii* (Ab12)	-	+
75	*A. baumannii* (Ab139)	-	-
76	*A. baumannii* (Ab37)	-	+
77	*A. baumannii* (Ab138)	-	-
78	*A. baumannii* (Abry5)	-	-
79	*A. baumannii* (Ab141)	-	-
80	*A. baumannii* (Ab14new)	-	-
81	*A. baumannii* (Abry149)	-	-
82	*A. baumannii* (Abry24)	-	+
83	*A. baumannii* (Ab39)	-	-
84	*A. baumannii* (Ab75)	-	-
85	*A. baumannii* (Ab27)	-	-
86	*A. baumannii* (Ab10)	-	-
87	*A. baumannii* (Ab3new)	-	-
88	*A. baumannii* (Abry6)	-	+
89	*A. baumannii* (Ab144)	-	+
90	*A. baumannii* (Abry9)	-	+

91	*A. baumannii* (Abry7)	-	-
92	*A. baumannii* (Ab20)	-	-
93	*A. baumannii* (Abry13)	-	+
94	*A. baumannii* (Ab10new)	-	+
95	*A. baumannii* (Abry12)	-	+
96	*A. baumannii* (Abry38)	-	-
97	*A. baumannii* (Ab128)	-	-
98	*A. baumannii* (Ab142)	-	+
99	*A. baumannii* (Abry14)	-	+
100	*A. baumannii* (Abry3)	-	

Supplementary Figure 1. Spot assay of *Acinetobacter baumannii* strains treated with imidazole. To eliminate potential interference from imidazole, present in the 31TSP solution, a control experiment was designed. The imidazole concentration in the obtained 31TSP solution was determined to be 100 mM. A series of imidazole solutions were prepared by diluting the 100 mM stock solution following the same dilution ratios used for 31TSP in this study. Subsequently, 5 μL aliquots of each diluted imidazole solution were spotted onto LB agar plates containing bacterial lawns, followed by incubation at 37°C for 24 h. The results are presented in the following. Blue numbers (0–8): Imidazole concentration gradient (0 = control, 1–8 = decreasing concentrations). Black numbers (bottom): Strain identifiers of *A. baumannii*.

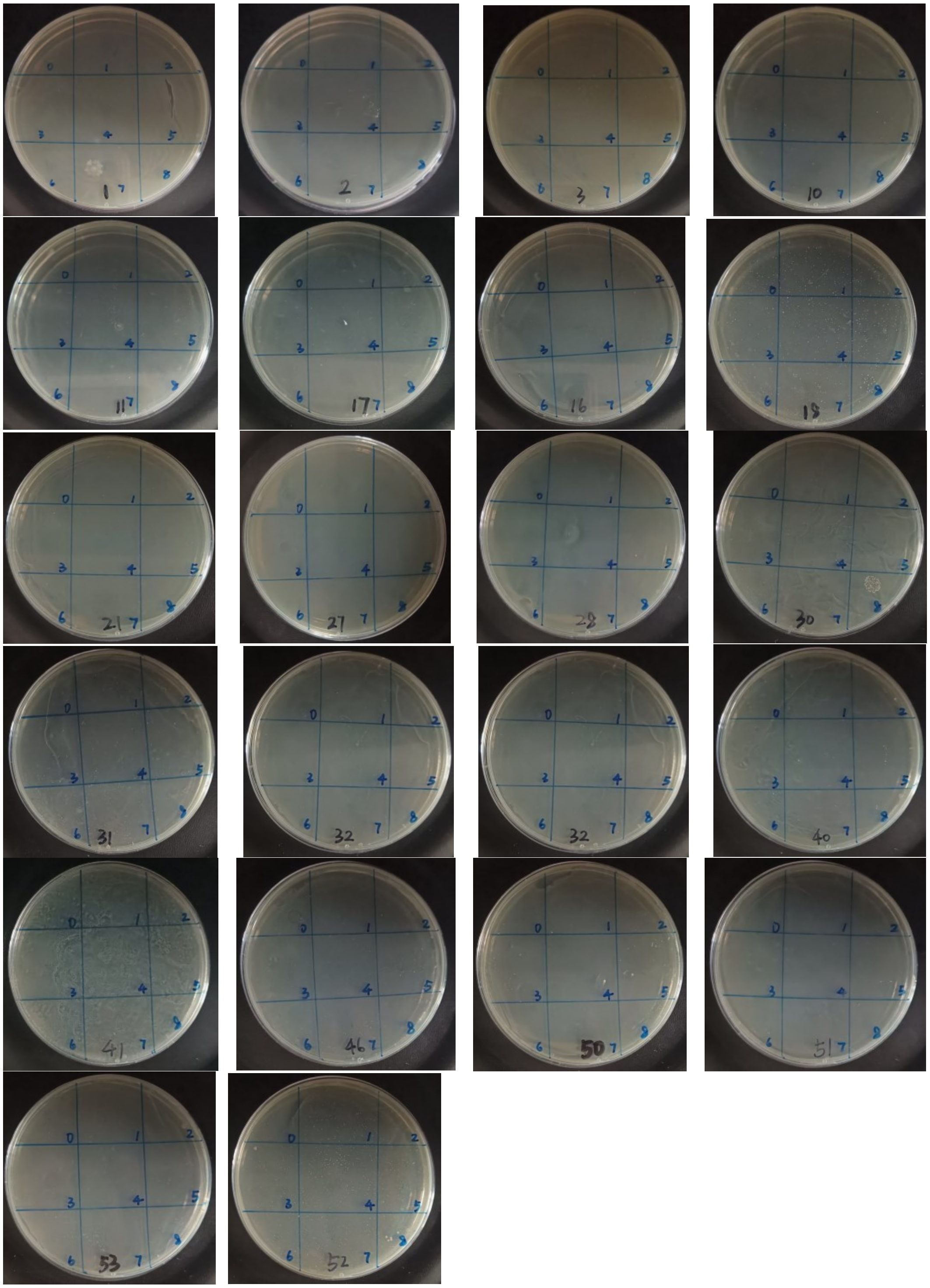
1
2
3
10
11
17
16
18
21
27
28
30
31
32
32
40
41
46
50
51
53
52

Supplementary Figure 2: Biofilm substrate growth curves of the host bacterium Ap31. The data are expressed as the absorbance at 570 nm of crystal violet staining of biofilm formation by host bacterium Ap31 at different time periods from 3-24 h. The amount of biofilm of Ap31 firstly increased with the change of time during 24 h, and then began to decrease when it reached the maximum value; the amount of biofilm was maximum when the incubation time was 9 h. The results are expressed as the mean ± SD (n = 3). Results are expressed as mean ± SD (n = 3)

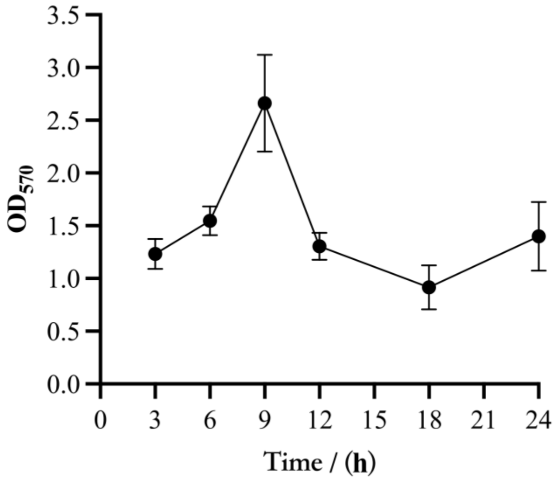

Supplement: Supplementary file 1 [file DataSheet1.pdf]
